# Thio­phene-2-carbonyl azide

**DOI:** 10.1107/S1600536813019740

**Published:** 2013-07-24

**Authors:** Gene C. Hsu, Laci M. Singer, David B. Cordes, Michael Findlater

**Affiliations:** a Department of Chemistry & Biochemistry, Texas Tech University, Memorial Circle & Boston, Lubbock, TX 79409, USA

## Abstract

The title compound, C_5_H_3_N_3_OS, is almost planar (r.m.s. deviation for the ten non-H atoms = 0.018 Å) and forms an extended layer structure in the (100) plane, held together *via* hydrogen-bonding inter­actions between adjacent mol­ecules. Of particular note is the occurrence of *R*C—H⋯N^−^=N^+^=N*R* inter­actions between an aromatic C—H group and an azide moiety which, in conjunction with a complementary C—H⋯O=C inter­action, forms a nine-membered ring.

## Related literature

For a previous preparation of the title compound, see: Binder *et al.* (1977 ▶). For the synthesis of the starting material, 2-thio­phene­carbonyl chloride, see: Kruse *et al.* (1989 ▶). For related structures, see: Arsenyan *et al.* (2008 ▶); Elshaarawy & Janiak (2011 ▶); Low *et al.* (2009 ▶).
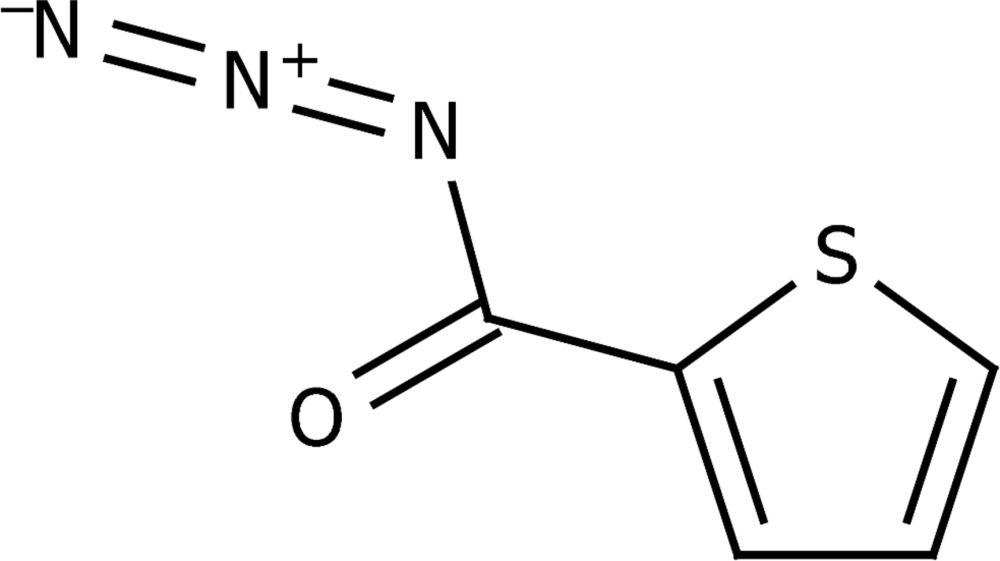


## Experimental

### 

#### Crystal data

C_5_H_3_N_3_OS
*M* *_r_* = 153.16
Monoclinic, 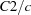

*a* = 12.668 (3) Å
*b* = 6.2153 (12) Å
*c* = 16.400 (3) Å
β = 95.91 (3)°
*V* = 1284.4 (4) Å^3^
*Z* = 8
Mo *K*α radiation
μ = 0.43 mm^−1^
*T* = 153 K
0.20 × 0.16 × 0.15 mm

#### Data collection

Nonius KappaCCD diffractometer
Absorption correction: multi-scan (*DENZO* and *SCALEPACK*; Otwinowski & Minor, 1997 ▶) *T*_min_ = 0.920, *T*_max_ = 0.939
2728 measured reflections
1459 independent reflections
1152 reflections with *I* > 2σ(*I*)
*R*_int_ = 0.025

#### Refinement

*R*[*F* ^2^ > 2σ(*F* ^2^)] = 0.050
*wR*(*F* ^2^) = 0.154
*S* = 1.13
1459 reflections
91 parameters
H-atom parameters constrained
Δρ_max_ = 0.59 e Å^−3^
Δρ_min_ = −0.49 e Å^−3^

### 

Data collection: *COLLECT* (Nonius, 1998 ▶); cell refinement: *COLLECT*; data reduction: *DENZO* and *SCALEPACK* (Otwinowski & Minor, 1997 ▶); program(s) used to solve structure: *SIR97* (Altomare *et al.*, 1999 ▶); program(s) used to refine structure: *SHELXL2013* (Sheldrick, 2013 ▶); molecular graphics: *SHELXTL* (Sheldrick, 2008 ▶) and *Mercury* (Macrae *et al.*, 2008 ▶); software used to prepare material for publication: *SHELXTL*, *enCIFer* (Allen *et al.*, 2004 ▶) and *publCIF* (Westrip, 2010 ▶).

## Supplementary Material

Crystal structure: contains datablock(s) global, I. DOI: 10.1107/S1600536813019740/tk5242sup1.cif

Structure factors: contains datablock(s) I. DOI: 10.1107/S1600536813019740/tk5242Isup2.hkl

Supplementary material file. DOI: 10.1107/S1600536813019740/tk5242Isup3.cml

Additional supplementary materials:  crystallographic information; 3D view; checkCIF report

## Figures and Tables

**Table 1:** Hydrogen-bond geometry (Å, °)

*D*—H⋯*A*	*D*—H	H⋯*A*	*D*⋯*A*	*D*—H⋯*A*
C2—H2⋯N1^i^	0.95	2.63	3.512 (4)	155
C3—H3⋯N3^ii^	0.95	2.66	3.396 (4)	135
C4—H4⋯O1^ii^	0.95	2.47	3.415 (4)	173

Symmetry codes: (i) 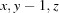; (ii) 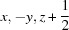.

**Fig. 1.**
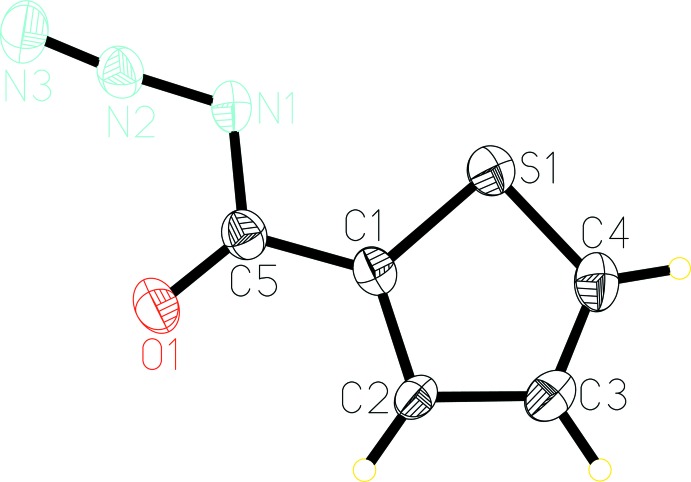
The molecular structure of the title compound with displacement ellipsoids drawn at the 50% probability level.

**Fig. 2.**
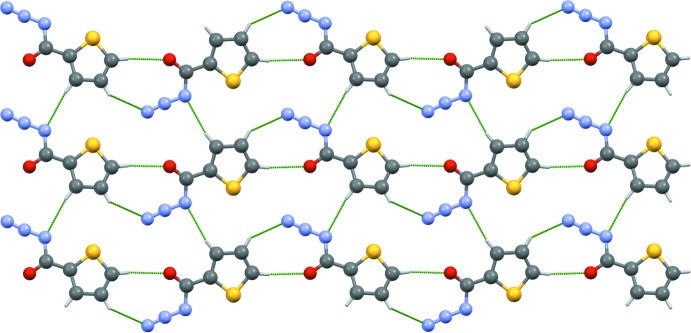
View of one of the hydrogen-bonded sheets in the (1 0 0) plane.

## supplementary crystallographic information

### Comment

The title compound (Fig. 1) displays a different organization in the solid state to that seen in related compounds. It forms one-dimensional hydrogen-bonded chains through the formation of C—H···N/O hydrogen bonds (Table 1 and Fig. 2), that are then linked into two-dimensional sheets in the (1 0 0) plane by further C—H···N interactions. This results in utilization of all the H atoms in the molecule for hydrogen-bonding. All three related structures (Arsenyan *et al.*, 2008; Elshaarawy & Janiak, 2011; Low *et al.*, 2009) are, in contrast, dominated by N—H···O/N hydrogen bonding, resulting in two different one-dimensional chains (Arsenyan *et al.*, 2008; Low *et al.*, 2009), and a two-dimensional sheet (Elshaarawy & Janiak, 2011).

### Experimental

The title compound was prepared by the method of Binder *et al.* (1977), from 2-thiophenecarbonyl chloride (Kruse *et al.*, 1989). Crystals suitable for X-ray structure determination were obtained by cooling a toluene solution of the title compound to -30°C.

### Refinement

Carbon-bound H atoms were included in calculated positions (C—H distances are 0.95 Å) and refined as riding atoms with *U*_iso_(H) = 1.2 *U*_eq_(parent atom).

### Crystal data

**Table:**

C_5_H_3_N_3_OS	*F*(000) = 624
*M**_r_* = 153.16	*D*_x_ = 1.584 Mg m^−^^3^
Monoclinic, *C*2/*c*	Mo *K*α radiation, λ = 0.71073 Å
*a* = 12.668 (3) Å	Cell parameters from 1494 reflections
*b* = 6.2153 (12) Å	θ = 1.0–27.5°
*c* = 16.400 (3) Å	µ = 0.43 mm^−^^1^
β = 95.91 (3)°	*T* = 153 K
*V* = 1284.4 (4) Å^3^	Block, colourless
*Z* = 8	0.20 × 0.16 × 0.15 mm

### Data collection

**Table:**

Nonius KappaCCD diffractometer	1152 reflections with *I* > 2σ(*I*)
Radiation source: fine focus sealed tube	*R*_int_ = 0.025
φ and ω scans	θ_max_ = 27.4°, θ_min_ = 2.5°
Absorption correction: multi-scan (*DENZO* and *SCALEPACK*; Otwinowski & Minor, 1997)	*h* = −15→16
*T*_min_ = 0.920, *T*_max_ = 0.939	*k* = −7→8
2728 measured reflections	*l* = −21→21
1459 independent reflections	

### Refinement

**Table:**

Refinement on *F*^2^	Primary atom site location: structure-invariant direct methods
Least-squares matrix: full	Secondary atom site location: difference Fourier map
*R*[*F*^2^ > 2σ(*F*^2^)] = 0.050	Hydrogen site location: inferred from neighbouring sites
*wR*(*F*^2^) = 0.154	H-atom parameters constrained
*S* = 1.13	*w* = 1/[σ^2^(*F*_o_^2^) + (0.0659*P*)^2^ + 3.253*P*] where *P* = (*F*_o_^2^ + 2*F*_c_^2^)/3
1459 reflections	(Δ/σ)_max_ = 0.001
91 parameters	Δρ_max_ = 0.59 e Å^−^^3^
0 restraints	Δρ_min_ = −0.49 e Å^−^^3^

### Special details

**Table:**

Geometry. All e.s.d.'s (except the e.s.d. in the dihedral angle between two l.s. planes) are estimated using the full covariance matrix. The cell e.s.d.'s are taken into account individually in the estimation of e.s.d.'s in distances, angles and torsion angles; correlations between e.s.d.'s in cell parameters are only used when they are defined by crystal symmetry. An approximate (isotropic) treatment of cell e.s.d.'s is used for estimating e.s.d.'s involving l.s. planes.

### Fractional atomic coordinates and isotropic or equivalent isotropic displacement parameters (Å^2^)

**Table:**

	*x*	*y*	*z*	*U*_iso_*/*U*_eq_	
C1	0.3676 (2)	0.0373 (4)	0.57926 (15)	0.0260 (6)	
C2	0.3494 (2)	−0.1827 (4)	0.59864 (15)	0.0238 (6)	
H2	0.3401	−0.2988	0.5609	0.029*	
C3	0.3478 (2)	−0.1956 (5)	0.68730 (18)	0.0334 (7)	
H3	0.3368	−0.3267	0.7150	0.040*	
C4	0.3632 (2)	−0.0048 (5)	0.72675 (17)	0.0347 (7)	
H4	0.3647	0.0105	0.7845	0.042*	
C5	0.3763 (2)	0.1133 (5)	0.49575 (15)	0.0271 (6)	
N1	0.3946 (2)	0.3386 (4)	0.49385 (13)	0.0323 (6)	
N2	0.39920 (19)	0.4093 (4)	0.42227 (13)	0.0308 (6)	
N3	0.4044 (2)	0.4879 (5)	0.36125 (15)	0.0401 (7)	
O1	0.36928 (17)	−0.0003 (4)	0.43538 (12)	0.0382 (5)	
S1	0.37950 (6)	0.20165 (12)	0.66320 (4)	0.0360 (3)	

### Atomic displacement parameters (Å^2^)

**Table:**

	*U*^11^	*U*^22^	*U*^33^	*U*^12^	*U*^13^	*U*^23^
C1	0.0249 (13)	0.0295 (14)	0.0237 (12)	−0.0011 (11)	0.0039 (10)	−0.0039 (10)
C2	0.0247 (13)	0.0226 (13)	0.0248 (12)	0.0005 (10)	0.0062 (10)	0.0053 (10)
C3	0.0358 (16)	0.0308 (16)	0.0340 (15)	−0.0005 (12)	0.0055 (12)	0.0079 (12)
C4	0.0364 (16)	0.0438 (18)	0.0240 (13)	0.0034 (14)	0.0035 (11)	0.0026 (12)
C5	0.0251 (13)	0.0311 (15)	0.0255 (13)	−0.0021 (11)	0.0045 (10)	−0.0028 (11)
N1	0.0438 (14)	0.0336 (13)	0.0196 (11)	−0.0007 (11)	0.0028 (9)	−0.0002 (9)
N2	0.0317 (13)	0.0335 (14)	0.0266 (12)	−0.0035 (10)	0.0002 (9)	−0.0026 (10)
N3	0.0461 (16)	0.0450 (16)	0.0286 (13)	−0.0109 (13)	0.0005 (10)	0.0039 (12)
O1	0.0519 (14)	0.0382 (12)	0.0255 (10)	−0.0086 (10)	0.0080 (8)	−0.0072 (9)
S1	0.0487 (5)	0.0318 (4)	0.0275 (4)	−0.0028 (3)	0.0036 (3)	−0.0018 (3)

### Geometric parameters (Å, º)

**Table:**

C1—C2	1.428 (4)	C4—S1	1.679 (3)
C1—C5	1.464 (4)	C4—H4	0.9500
C1—S1	1.708 (3)	C5—O1	1.212 (3)
C2—C3	1.459 (4)	C5—N1	1.420 (4)
C2—H2	0.9500	N1—N2	1.260 (3)
C3—C4	1.355 (4)	N2—N3	1.122 (3)
C3—H3	0.9500		
			
C2—C1—C5	123.1 (2)	C3—C4—S1	113.2 (2)
C2—C1—S1	113.34 (19)	C3—C4—H4	123.4
C5—C1—S1	123.5 (2)	S1—C4—H4	123.4
C1—C2—C3	107.1 (2)	O1—C5—N1	123.7 (2)
C1—C2—H2	126.5	O1—C5—C1	124.8 (3)
C3—C2—H2	126.5	N1—C5—C1	111.5 (2)
C4—C3—C2	114.3 (3)	N2—N1—C5	112.8 (2)
C4—C3—H3	122.8	N3—N2—N1	174.5 (3)
C2—C3—H3	122.8	C4—S1—C1	92.11 (14)
			
C5—C1—C2—C3	178.9 (2)	S1—C1—C5—N1	−0.6 (3)
S1—C1—C2—C3	−0.5 (3)	O1—C5—N1—N2	2.1 (4)
C1—C2—C3—C4	0.0 (3)	C1—C5—N1—N2	−178.0 (2)
C2—C3—C4—S1	0.5 (4)	C3—C4—S1—C1	−0.7 (3)
C2—C1—C5—O1	−0.1 (4)	C2—C1—S1—C4	0.7 (2)
S1—C1—C5—O1	179.2 (2)	C5—C1—S1—C4	−178.7 (2)
C2—C1—C5—N1	−179.9 (2)		

### Hydrogen-bond geometry (Å, º)

**Table:**

*D*—H···*A*	*D*—H	H···*A*	*D*···*A*	*D*—H···*A*
C2—H2···N1^i^	0.95	2.63	3.512 (4)	155
C3—H3···N3^ii^	0.95	2.66	3.396 (4)	135
C4—H4···O1^ii^	0.95	2.47	3.415 (4)	173

Symmetry codes: (i) *x*, *y*−1, *z*; (ii) *x*, −*y*, *z*+1/2.

## References

[bb1] Allen, F. H., Johnson, O., Shields, G. P., Smith, B. R. & Towler, M. (2004). *J. Appl. Cryst.* **37**, 335–338.

[bb2] Altomare, A., Burla, M. C., Camalli, M., Cascarano, G. L., Giacovazzo, C., Guagliardi, A., Moliterni, A. G. G., Polidori, G. & Spagna, R. (1999). *J. Appl. Cryst.* **32**, 115–119.

[bb3] Arsenyan, P., Petrenko, A. & Belyakov, S. (2008). *Tetrahedron Lett.* **49**, 5255–5257.

[bb4] Binder, D., Habison, G. & Noe, C. R. (1977). *Synthesis*, pp. 255–256.

[bb5] Elshaarawy, R. F. & Janiak, C. (2011). *Z. Naturforsch. Teil B*, **66**, 1201–1208.

[bb6] Kruse, L. I., Ladd, D. L., Harrsch, P. B., McCabe, F. L., Mong, S.-M., Faucette, L. & Johnson, R. (1989). *J. Med. Chem.* **32**, 409–417. 10.1021/jm00122a020 2913301

[bb7] Low, J. N., Quesada, A., Santos, L. M. N. B. F., Schröder, B. & Gomes, L. R. (2009). *J. Chem. Crystallogr.* **39**, 747–752.

[bb8] Macrae, C. F., Bruno, I. J., Chisholm, J. A., Edgington, P. R., McCabe, P., Pidcock, E., Rodriguez-Monge, L., Taylor, R., van de Streek, J. & Wood, P. A. (2008). *J. Appl. Cryst.* **41**, 466–470.

[bb9] Nonius (1998). *COLLECT* Nonius BV, Delft, The Netherlands.

[bb10] Otwinowski, Z. & Minor, W. (1997). *Methods in Enzymology*, Vol. 276, *Macromolecular Crystallography*, Part A, edited by C. W. Carter Jr & R. M. Sweet, pp. 307–326. New York: Academic Press.

[bb11] Sheldrick, G. M. (2008). *Acta Cryst.* A**64**, 112–122. 10.1107/S0108767307043930 18156677

[bb12] Sheldrick, G. M. (2013). *SHELX2013* University of Göttingen, Germany.

[bb13] Westrip, S. P. (2010). *J. Appl. Cryst.* **43**, 920–925.

